# Deaths from novel psychoactive substances in England, Wales and Northern Ireland: Evaluating the impact of the UK psychoactive substances act 2016

**DOI:** 10.1177/02698811211026645

**Published:** 2021-06-29

**Authors:** Adrian A Deen, Hugh Claridge, Richard D Treble, Hilary J Hamnett, Caroline S Copeland

**Affiliations:** 1 Institute of Pharmaceutical Sciences, King’s College London, London, United Kingdom; 2 Population Health Research Institute, St George’s, University of London, London, United Kingdom; 3 No affiliation (retired); 4 School of Chemistry, University of Lincoln, Lincoln, United Kingdom

**Keywords:** Novel psychoactive substance, substance misuse, psychoactive substances act, legal highs, drug-related death, misuse of drugs act, drug policy, designer drugs

## Abstract

**Background:**

‘Legal highs’ began appearing in the UK in the mid-2000s. Whilst many of these substances were controlled under the 1971 Misuse of Drugs Act, novel compounds and new variants of controlled compounds were continuously being introduced to the recreational drug market. The Psychoactive Substances Act (PSA) was therefore implemented in 2016 as a blanket ban on all novel psychoactive substances (NPS).

**Aim:**

To evaluate the impact of the PSA on deaths following NPS use in England, Wales and Northern Ireland.

**Methods:**

Cases reported to the National Programme on Substance Abuse Deaths where death had occurred 3 years pre- or post-implementation of the PSA were extracted. Cases with NPS detected at post-mortem were analysed and compared against cases non-NPS cases.

**Results:**

293 deaths with NPS detected were identified; 91 occurring before the PSA and 202 afterwards, indicating an 222.0% post-PSA increase. Contrastingly, non-NPS drug-related death case reporting increased by only 8.0%. Synthetic cannabinoid, anxiolytic/sedative and stimulant NPS were detected in the largest proportions of deaths pre-PSA; post-PSA stimulant NPS detections reduced whilst synthetic cannabinoid and anxiolytic/sedative detections increased.

Post-PSA, average decedent age increased significantly (mean age pre-PSA 34.4 ± 10.8 vs post-PSA 38.3 ± 9.4), and they were significantly more likely to have been living in deprived areas (pre-PSA 50.0% vs post-PSA 65.9%).

**Conclusions:**

Reporting of deaths following NPS use has risen despite introduction of the PSA. Whilst deaths amongst younger individuals and those living in more affluent areas has reduced, additional approaches to prohibition are needed to curb their persistence in deprived demographics.

## Introduction

### The Psychoactive Substances Act 2016

‘Legal highs’, which began appearing in the UK in the mid-2000s, were aimed at a niche middle class demographic of experimental users (‘psychonauts’) interested in exploring recreational drug diversity (Peacock et al., 2019). Indeed, they were especially popular amongst young people who – at this point – were able to legitimately purchase them online and from local ‘head shops’ – establishments specialising in the sale of legal recreational drugs and paraphernalia (Pillay and Kelly, 2010). The appeal of these substances over more traditional drugs of abuse appears to have stemmed from their legal status, that they did not appear on standard drug tests, and were cheap and readily available (Bonar et al., 2014; Brunt et al., 2017; Mathews et al., 2019; Weinstein et al., 2017). The UK Government sought to reduce the rate of use of these ‘legal highs’, consequentially implementing the UK Psychoactive Substances Act (PSA), which came into effect on May 26^th^ 2016 (UK-Government, 2016). The PSA was designed to ‘prohibit the distribution of non-controlled novel psychoactive substances’ (NPS), making the manufacture and supply of all NPS that hitherto had been legal, a punishable offence (UK-Government, 2016). The PSA was motivated by the belief that prohibition of NPS would reduce the health-related harms thought to be associated with them and curtail the efforts of new and emerging drug dealers (UK-Government, 2016). Prior to the PSA, illicit psychoactive substances were controlled individually under the Misuse of Drugs Act (MDA), 1971 (UK-Government, 1971). A labour-intensive and time-consuming process, the banning of substances under the MDA was based on the molecular structure of substances and the evidenced harms that these chemicals pose (UK-Government, 1971). In the time it took for the Advisory Council on the Misuse of Drugs (ACMD) to prepare evidence to support new MDA controls, underground chemists were already at work making small but significant changes to the molecular structure of these drugs to create new compounds that circumvented these controls (ACMD, 2011; Nutt, 2020; UK-Government, 1971). In an effort to address this, temporary class drug orders (TCDOs) were introduced in November 2011 whereby NPS causing sufficient concern for potential harms could be temporarily controlled under the MDA whilst evidence was being gathered. However, TCDOs still required identification of specific compounds and preliminary evidence of their potential harms (UK Home Office, 2011). Therefore, the PSA has largely replaced the issuance of TCDOs, and works together with the MDA in concert, with the PSA acting as the immediate prohibitive legislature for NPS manufacture and distribution whilst the required evidence for their banning under the MDA is collected. The maximum penalties under the PSA are generally more lenient than those of the MDA (and TCDOs, which follow MDA penalties) (UK Home Office, 2011). Indeed, whilst the PSA came under criticism when first introduced for its loose definition of psychoactive substances (see ‘Novel Psychoactive Substances’ below; (ACMD, 2015), which could be interpreted as banning, amongst other things, flowers, perfume and the use of incense in churches (Dunt, 2015), it was praised by drug policy reformers for not criminalising possession of NPS for personal consumption (Transform, 2021). This was seen by some lobbyists as a positive step towards the ‘Portugal model’ of decriminalising possession whilst keeping supply illegal (Cowan, 1986; Félix and Portugal, 2017). However, with the closure of ‘head shops’, the sourcing of NPS switched to street dealers and the darknet (Deligianni et al., 2020), both which carry their own risks: The former exposes NPS users to dealers who want to sell more dangerous other drugs, and the latter makes users potentially prosecutable under the PSA as purchase of NPS online (Deligianni et al., 2020; Miliano et al., 2018), even if intended for personal use, could be classed as import. Whilst there have been successful prosecutions made under the PSA, debate around whether a substance can be classed as an NPS (for example, whether it has direct or indirect effects on the central nervous system (Fortson, 2018) has elongated case proceedings demonstrating high complexity in its implementation and concomitant financial burden.

### Novel psychoactive substances

The ACMD first used the ‘NPS’ term in their 2011 report on ‘legal highs’. They defined NPS as: ‘Psychoactive substances which are not prohibited by the United Nations Single Convention on Narcotic Drugs or by the MDA, 1971, and which people in the UK are seeking for intoxicant use’ (ACMD, 2011). Although aspects of this definition informed much of the thinking behind the PSA legislation, the Act does not explicitly preface the banning of psychoactive substances with the word ‘novel’ (Mdege et al., 2017). Instead, the PSA adopted a much broader banning of: ‘All substances that act to stimulate or depress brain function’ (UK-Government, 2016). With the exception of foods, alcohol and psychoactive substances used for medicinal purposes, a vast number of drugs were made subject to the provisions of the Act (UK-Government, 2016). An all-encompassing definition, the PSA was intended to ensure that no newly made or newly repurposed drugs escaped legislative control.

### PSA and NPS

The UK remains one of the biggest consumers of NPS in Europe (Global-Drugs-Survey, 2019). Given this, the introduction of the PSA has instigated research into its efficacy as a deterrent for NPS-taking behaviours (Reuter and Pardo, 2017). Deligianni et al. (2020) recently published survey results on the impact of the PSA on people’s use and awareness of health risks associated with NPS. Self-reporting from 894 participants revealed an increase in use of NPS amongst the sample group along with a downwards trend in respondent’s awareness of associated health risks (Deligianni et al., 2020), findings in line with that of the Home Office’s own assessment in 2018 (UK Home Office, 2018). They conclude that a more systematic approach is needed to assess the effectiveness of the PSA as the results from their study revealed no significant change in attitudes to NPS use since its introduction (Deligianni et al., 2020).

As yet, there has been no systematic analysis of drug-related deaths (DRDs) before and after the introduction of the PSA. A systematic analysis will produce a much more conclusive picture of the impact of the PSA on public health – a supposition in keeping with a long history of using DRDs as an objective metric for the potential harm of drugs (Corkery et al., 2020). In this paper we look at DRDs from England, Wales and Northern Ireland in which NPS were detected at post-mortem in the 3 years pre- and post-introduction of the PSA. Our analysis has revealed an overall increase in NPS DRD reporting since the introduction of the PSA in 2016. Based on toxicology reports submitted to the National Programme on Substance Abuse Deaths (NPSAD) by coroners, our research allows for commentary on the impact of the PSA and in turn broader UK drug legislation. Our results underscore the debate around banning drugs versus regulating them and postulate on the effect the PSA has had on other drug-taking behaviours. This research aims to add to the growing evidence-base on NPS in order to better inform policy and achieve NPS harm reduction.

## Method

### National programme on substance abuse deaths

Data were collated from case reports submitted to NPSAD, which receives regular voluntary coroner’s reports on DRDs from 75 of the 93 coronial jurisdictions (80.6%) in England, Wales and Northern Ireland. Reports were previously received from the Scottish Crime and Drug Enforcement Agency, but these ceased in 2011. A death is deemed drug-related by coroners where drugs were considered contributory to the death occurring. Cases include deaths from prescription medications, recreational drugs, NPS and intravenous drug use. Coroners investigate deaths resulting from a range of causes deemed to be unnatural; this includes violent and sudden deaths, unexplained deaths, deaths that occur before a patient comes out of anaesthetic as well as deaths caused by industrial disease or poisoning. Toxicology tests are requested dependent upon individual case circumstances and at the discretion of the coroner. The average time between death and conclusion of coronial inquest, which is when cases are reported to NPSAD, is 7.2 months.

The King’s College London Biomedical and Health Sciences, Dentistry, Medicine and Natural and Mathematical Sciences Research Ethics SubCommittee (BDM RESC) confirmed in November 2020 that NPSAD does not require REC review as all subjects are deceased. Neither the General Data Protection Regulation nor the Data Protection Act apply to identifiable data that relate to a person once they have died. Nevertheless, personal data of deceased individuals were treated with the strictest confidentiality and anonymised for analysis purposes.

### Case Identification

NPS were defined as psychoactive compounds not under the control of the MDA or a TCDO prior to May 26th 2016. Cases where NPS were administered prior to death were identified by searching the post-mortem drug fields for mention of all NPS detected in cases reported to NPSAD. All cases contained toxicology evidence confirming the presence of NPS metabolites and/or parent molecules in decedents’ post-mortem tissue(s). Toxicological evidence and drug-related coronial conclusions were used as the criteria for defining an NPS case rather than cause(s) of death, as it is not uncommon for ambiguous drug-related causes to be cited (e.g. multi-drug toxicity, polydrug abuse), or environmental factors that caused death as a result of drug use (e.g. fall from a height) to be listed.

### Case analysis

IBM® SPSS software (version 25) was used for case extraction and analysis. All cases reported to NPSAD as of September 1st 2020 where death had occurred during the 6 year period (26th May 2013–25th May 2019) were extracted. It is expected that the vast majority of qualifying cases will have been reported to NPSAD at time of writing, as over 15 months (i.e. twice the usual time between death and conclusion of coronial inquest) had elapsed between the end of the study period and date of data collection. Cases were then categorised as NPS or non-NPS cases dependent upon whether or not NPS were detected in post-mortem tissue(s) according to Home Office publications on MDA and PSA controlled drugs (UK government, 1971). Cases were then further categorised into deaths that occurred in the 3 year period before the introduction of the PSA (May 26th 2013–May 25th 2016) and those that occurred afterwards (May 26th 2016–May 25th 2019).

Statistical tests (Student’s t test, Chi Squared) were performed using IBM® SPSS software (version 25).

Deprivation deciles were determined by postcode matching the usual address of decedents with the English, Welsh and Northern Irish Indices of Deprivation calculators.

## Results

As of September 1st, 2020, 11,253 deaths had been reported to NPSAD that had occurred between 26th May 2013 and 25th May 2019. In 293 of these deaths (2.6% of all cases) NPS were detected, with a total of 363 individual NPS detections made from these cases (i.e. in some cases multiple NPS were detected). Of these 293 deaths where NPS were detected, 91 occurred in the 3 years prior to implementation of the PSA (31.1%), with 202 afterwards (69.9%), representing a 222.0% increase in deaths with NPS detections following introduction of the PSA. By comparison, the overall number of non-NPS DRDs reported to NPSAD increased by only 8.0% (5269 deaths pre-PSA, 5691 deaths post-PSA). Furthermore, when normalised against total NPSAD reporting over the same time period to account for fluctuations in raw NPSAD reporting figures, the increase in deaths with NPS detected remains, demonstrating that there has been a proportional rise in their occurrence (data not shown). 32 different NPS were detected: nine still subject to the PSA at the time of writing, with the other 23 drugs having been subsequently specifically controlled under the MDA. In 96.6% of cases (*n* = 283/293) drug use was cited as a cause of death, with 84.5% of cases (*n* = 239/283) specifically citing the NPS.

### Types of NPS

**Figure 1.**
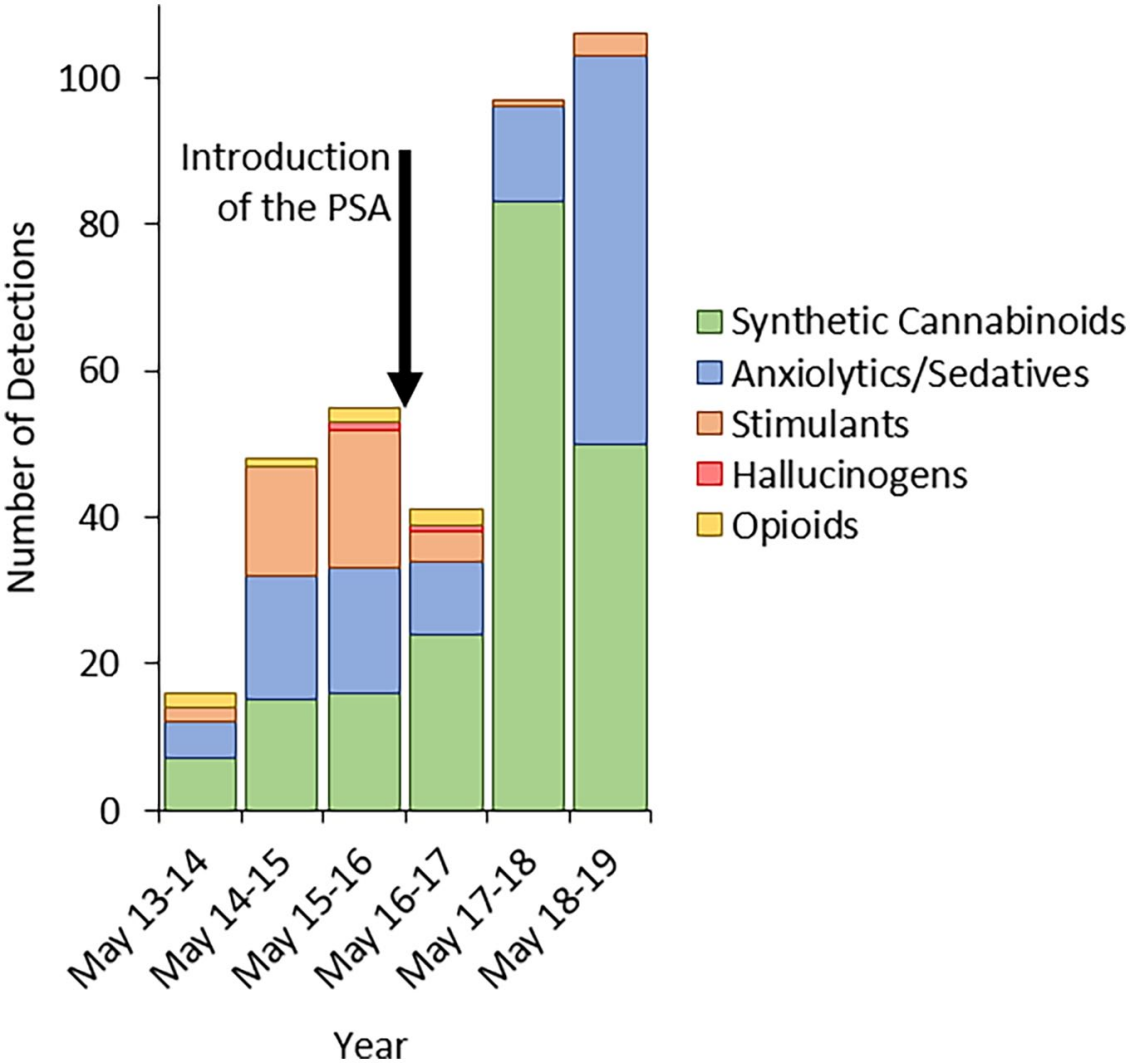
Detections by NPS type in cases reported to NPSAD from England, Wales and Northern Ireland where death occurred between May 26th 2013 and May 25th 2019. Year periods are between May 26th and May 25th.

NPS were categorised by their chemical structure and pharmacology in accordance with the European Monitoring Centre for Drugs and Drug Addiction descriptions as synthetic cannabinoid receptor agonists (SCRAs), stimulants, hallucinogens, opioids or anxiolytic/sedatives (Table 1). Detections of hallucinogens (0.6% of detections, *n* = 2/363) and opioids (1.9% of detections, *n* = 7/363) comprised a small proportion of all NPS detections (Figure 1; Table 1). SCRAs (53.7% of detections, *n* = 195/363), anxiolytic/sedatives (31.7% of detections, *n* = 115/363) and stimulants (12.1% of detections, *n* = 44/363) formed a much greater proportion of total detections, with deaths positive for SCRAs and anxiolytics/sedatives having risen, and those involving stimulants having fallen since the introduction of the PSA (Figure 1; Table 1). Whilst the rise in deaths with SCRAs detected post-PSA can be mainly attributed to increased detections of the compounds 5F-MDMB-PINACA (1.0% of pre-PSA detections; 22.6% of post-PSA detections) and AB-FUBINACA (0.5% of pre-PSA detections; 9.9% of post-PSA detections), there were reductions in detections of other SCRA compounds, such as 5F-APINACA (2.5% of pre-PSA detections; 0.5% of post-PSA detections) and 5F-QUPIC (2.0% of pre-PSA detections; 0.8% of post-PSA detections). Similarly, the increase in anxiolytic/sedatives detections can be majority attributed to a single anxiolytic compound – etizolam (4.3% of pre-PSA detections; 15.3% of post-PSA detections). The fall in deaths with stimulants detected post-PSA can be majority attributed to decreased detections of methoxphenidine (5.6% of pre-PSA detections; 0.3% of post-PSA detections).

**Table 1.** NPS detections by drug class pre- and post-introduction of the PSA where death occurred between May 26th 2013 and May 25th 2019.

Drug class	NPS	Number of deaths	Number of deaths
		Pre-PSA	Post-PSA
Synthetic cannabinoids		38	157
Initially PSA, now MDA	4F-MDMB-BINACA	0	6
	5F-AMB	0	1
	5F-APICA	3	0
	5F-APINACA	10	2
	5F-MDMB-PICA	0	5
	5F-MDMB-PINACA	4	89
	5F-MMB-PICA	0	2
	5F-QUPIC	8	3
	AB-CHIMINACA	3	0
	AB-FUBINACA^	2	39
	AB-PINACA	1	0
	APP-BINACA	0	1
	MDMB-4en-PINACA	0	1
	MDMB-CHMICA	6	6
	MMB-CHMICA	0	2
	QUCHIC	1	0
Anxiolytics/sedatives		39	76
*PSA Controlled*	Flualprazolam	0	4
*Initially PSA, now MDA*	Diclazepam	6	8
	Etizolam	17	60
	Flubromazepam	13	3
	Flubromazolam	2	0
	Pyrazolam	1	1
Stimulants		36	8
PSA controlled	2-AI	1	2
	1,2-Diphenidine	4	0
	3-FPM	7	3
	5-IAI	1	1
	Methoxphenidine	23	1
Initially PSA, now MDA	4-Fluoromethylphenidate	0	1
Opioids		5	2
PSA controlled	Kratom	5	1
Initially PSA, now MDA	U47700	0	1
Hallucinogens		1	1
PSA controlled	Methoxypiperamide	1	1

### Control status

14.0% (*n* = 55/363) of NPS detections were of NPS still controlled under the PSA at time of writing. 76.4% of these detections (*n* = 42/55) occurred in the 3 years prior to the introduction of the PSA, with 23.6% (*n* = 13/55) occurring afterwards.

NPS drugs that initially were subject to the PSA when it was first introduced but have subsequently been specifically controlled by the MDA account for the largest proportion of NPS detections (86.0%, *n* = 338/363). Of the 338 detections in this category, 22.8% (*n* = 77/338) occurred before the introduction of the PSA with 77.2% (*n* = 261/338) occurring afterwards.

### Deaths from established MDA-controlled drugs

DRDs relating to MDMA, cocaine and the benzodiazepine alprazolam are of particular interest as they each contributed to more deaths in the 3 years following introduction of the PSA, in comparison to the 3 years prior to its introduction (MDMA 160 deaths pre-PSA, 210 deaths post-PSA; cocaine 1346 deaths pre-PSA, 2393 deaths post-PSA; alprazolam 27 deaths pre-PSA, 318 deaths post-PSA). Whilst this is not an extensive list of more commonly used drugs, our interest in them has emerged from the Home Office’s published report on the potential displacement of PSA banned NPS with more traditionally used substances (UK Home Office, 2018). The 77.8% post-PSA increase in deaths for which cocaine was detected at post-mortem is especially note-worthy in light of the 77.8% drop in DRDs where novel stimulants were detected since the PSA was introduced (Figure 1; Table 1).

### Demographics

**Figure 2.**
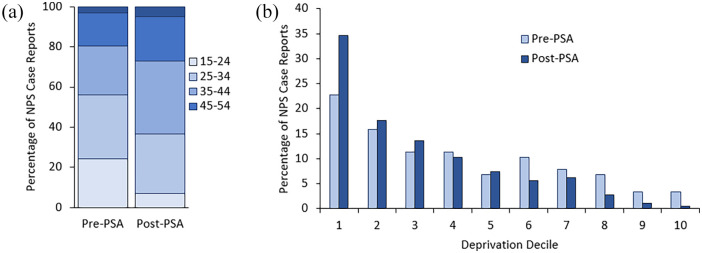
(a) Percentage of NPS cases by age range, and (b) deprivation decile by postcode of usual address of decedents with NPS detected at post-mortem, pre- and post-introduction of the PSA reported to NPSAD from England, Wales and Northern Ireland where death occurred between May 26th 2013 and May 25th 2019.

For both NPS and non-NPS cases, males accounted for a significant majority of deaths pre- and post-PSA (Table 1). Furthermore, NPS cases were significantly more likely to be male than non-NPS cases (87.7% vs 72.0%; *p* < 0.01). In cases with NPS detected, decedents were significantly older at time of death (*p* < 0.1) in the post-PSA period whereas the average age at time of death for non-NPS decedents remained unchanged (Table 2; Figure 2(a)). Decedents who died following NPS administration after the introduction of the PSA were – compared to those who died before the Act was introduced – significantly more likely to be from the most deprived areas of the UK (deprivation deciles 1-3; pre-PSA 50.0% vs post-PSA 65.9%; *p* < 0.1) (Figure 2(b)). Furthermore, the proportion of decedents where NPS were detected who were living in private residential accommodation significantly reduced (Table 2, *p* < 0.01), and those listed as homeless, living in a hostel or residing in prison significantly rose, following introduction of the PSA (Table 2; *p* < 0.01). Finally, the proportion of decedents with no prior history of drug abuse significantly reduced following introduction of the PSA (20.9% pre-PSA, *n* = 19/91; 6.9% post-PSA, *n* = 14/202).

**Table 2.** Gender, age and usual living circumstances of decedents in cases where NPS were and were not detected and reported to NPSAD from England, Wales and Northern Ireland where death occurred between May 26th 2013 and May 25th 2019.

	NPS cases		Non-NPS cases	
	Pre-PSA	Post-PSA	Pre-PSA	Post-PSA
Gender				
Men	90.1% (*n* = 82)	86.6% (*n* = 175)	71.8% (*n* = 3783)	72.2% (*n* = 4108)
Women	9.9% (*n* = 9)	13.4% (*n* = 27)	28.3% (*n* = 1483)	27.8% (*n* = 1583)
Mean age	34.4 ± 10.8	38.3 ± 9.4	42.1 ± 12.5	42.7 ± 12.8
Usual living circumstances				
Private residential	94.5% (*n* = 86)	74.8% (*n* = 151)	93.3% (*n* = 4919)	92.21% (*n* = 5247)
Hostel	3.3% (*n* = 3)	5.9% (*n* = 12)	1.9% (*n* = 99)	2.0% (*n* = 111)
Homeless	2.2% (*n* = 2)	11.9% (*n* = 24)	3.0% (*n* = 160)	3.9% (*n* = 223)
Prison	–	3.5% (*n* = 7)	0.1% (*n* = 6)	0.1% (*n* = 7)
Unknown	–	1.0% (*n* = 2)	0.1% (*n* = 7)	0.3% (*n* = 15)
Other^	–	3.0% (*n* = 6)	1.5% (*n* = 79)	1.5% (*n* = 87)

^ Other: Rehab, Hotel, Nursing Home, Hospital, Boat, Business Address, Motor Vehicle, Caravan.

## Discussion

Our results complement the 2018 Home Office review of the PSA, which found NPS to constitute a small proportion (4.7%) of total drug use in England and Wales (UK Home Office, 2018). The Home Office intended the PSA to dissuade individuals – especially young people – from using NPS; it also hoped to reduce health and social harms associated with these substances (Al-Banaa et al., 2020). Whilst fluctuations in NPS use since the introduction of the PSA allow for some commentary on the efficacy of the policy, a more objective assessment can be reached through a comparative analysis of NPS-associated fatality before and after the Act was brought in. Corkery et al. argue that DRDs are the most important metric for potential drug-associated harm; till now however no such comprehensive evaluation of the Act’s impact on NPS-associated fatality has been published (Corkery et al., 2018; Hill, 2020).

Whilst we show an increase in DRDs positive for NPS since the introduction of the PSA, the majority of the deaths with NPS detected occurring in the post-PSA period are of NPS that have since been specifically controlled under the MDA. This indicates that the proactive PSA is indeed controlling harmful NPS whilst the required evidence for their subsequent reactive control by the MDA is gathered. However, neither the PSA nor the MDA appear to be deterring NPS use that precedes death.

### Skewed by SCRAs

SCRAs comprise the majority of NPS detections. Incidences of death following SCRA use – both from SCRAs deemed to be NPS by this study and SCRAs specifically controlled under the MDA prior to implementation of the PSA – have dramatically increased in recent years, with no evidence of impact of the PSA on their reporting rates (Yoganathan et al., 2021). This apparent lack of relationship between the PSA and SCRA fatality rates requires further research, particularly with regards to the development of a more appropriate service response rather than further legislative change.

Motivations for SCRA use do not appear to derive from the enjoyment of their effects; conversely, SCRA users have indicated a preference for herbal cannabis as SCRAs are cited to elicit negative effects (Castaneto et al., 2014; Smith and Staton, 2019). Rather, SCRA use prior to the PSA appears to have been driven by their legal status, that standard drug tests do not include those that can detect SCRAs, and that they were cheap and readily available (Bonar et al., 2014; Brunt et al., 2017; Gunderson et al., 2014; Mathews et al., 2019; Scourfield et al., 2019; Weinstein et al., 2017). Indeed, following the control of many SCRA compounds as class B substances under the MDA or under the PSA, SCRA use in the general population was observed to decline (Blackman and Bradley, 2017). However, significant prevalence in some vulnerable sub-groups remains, particularly homeless individuals and those imprisoned who continue to use SCRAs due to their accessibility and difficulty in analytical detection (Blackman and Bradley, 2017; Brunt et al., 2017; Felvinczi et al., 2020; Ford and Berg, 2018; Norman et al., 2020; Peacock et al., 2019; Scourfield et al., 2019; Weinstein et al., 2017). Indeed, a major driver for SCRA use is their lack of odour during consumption, and lack of appearance on standard drug screens – factors that are well documented in the use of cannabis itself (Gray et al., 2021). Furthermore, SCRAs are reportedly both cheaper and more readily accessible than cannabis, with SCRA dealers actively approaching users, negating even the need to seek them out (Gray et al., 2021). SCRA use also appeals to these individuals due to their strongly intoxicating effects: they have been described to provide release from insufferable circumstances by enabling disengagement with reality (Blackman and Bradley, 2017; Csák et al., 2020; Ellsworth, 2019; Gray et al., 2020).

There is a constantly shifting pattern of SCRAs that are dominant within the NPS market (Castaneto et al., 2014; Wedinos, 2019). The SCRAs that are most abundant at any particular time reflect legal changes, not just within the UK, but internationally and particularly in China, the major producing country (Norman et al., 2020). However, reports of deaths where SCRAs were detected to NPSAD are projected to persist at a rate of ~50 deaths per year, indicating the need for alternate intervention approaches (Yoganathan et al., 2021). A ban citing commonly used names for SCRA preparations (e.g. ‘Spice’, ‘K2’, ‘Kronic’ and ‘Mamba’) as opposed to specific SCRA molecular structural variants may prove more effective, as was observed in Australia (Cairns et al., 2017).

### Displacing and replacing NPS

Prior to the PSA, Moore et al. carried out research into whether NPS displace, supplement or act as gate-way drugs for established drug use (Moore et al., 2013). They found in the case of the now-MDA-controlled mephedrone, that it was used to supplement rather than displace or replace other established stimulants like cocaine and ecstasy (Moore et al., 2013). Our results show a fall in NPS stimulant detections, but a rise in deaths involving established stimulants such as cocaine and MDMA in the period after introduction of the PSA. The higher cost of traditional drugs of abuse compared to NPS drugs was found to be one of the primary motivations for some NPS use prior to the PSA – as such these NPS served to displace more expensive established drugs (Deligianni et al., 2020; Smith and Garlich, 2013). Despite Moore et al.’s findings, this was found to be especially true for some party goers who took mephedrone (2011). Post-PSA the fall of NPS stimulant but rise in MDMA and cocaine deaths is multi-factorial, with MDMA having become more readily available, and cocaine having become both cheaper and purer over the 6 year study period likely having impact upon their more widespread use (Corkery et al., 2017; Rice et al., 2020). That said, evidence of their displacement by analogous NPS before the PSA, as well as our results showing the increase in DRDs from MDMA and cocaine since these analogues became banned, points to the potential for the PSA to have contributed to users turning or returning to established stimulants.

Our analysis also indicates a resurgence in deaths with detections of the NPS benzodiazepines flualprazolam and etizolam. This complements research published by McNamara et al. on the increased use of these benzodiazepines in vulnerable populations in Ireland (Mc Namara et al., 2019), a trend which has also emerged on a global scale (Nielsen and McAuley, 2020). The lower number of deaths involving other NPS anxiolytic/sedatives may – like the established stimulants – be a case of anxiolytic/sedative NPS use being displaced by increasingly available MDA controlled benzodiazepines, such as alprazolam (Hockenhull et al., 2019) and indeed etizolam itself – the latter both prior to and after its control under the MDA in May 2017.

### A devolving demographic

Like almost all DRDs in the UK, deaths with NPS detected are most prevalent amongst males under the age of 45 (Corkery et al., 2014). Specific to the potential impact of the PSA, decedents were on average older and more likely to have been residing in the most deprived areas of the UK or even homeless after introduction of the PSA. This may be due to the evolving reputation of NPS: The young middle class demographic of experimental users (‘psychonauts’) interested in exploring recreational drug diversity originally encouraged NPS use on online discussion forums but now actively deter others from their use (Bilgrei, 2016; Peacock et al., 2019). This may also be a driving factor for the decreasing trend in deaths in individuals who did not have an established history of substance misuse. This demographic shift may also be contributed to by the impact the PSA has had on how NPS are now supplied and sold (Smyth et al., 2020). The closing of ‘head shops’ drove the NPS market underground and as such into the hands of street dealers (Stevens et al., 2015). Street drug dealers largely operate in the most deprived areas of the country, also home to the most vulnerable populations (Lupton et al., 2002). Whilst there is no evidence to suggest the sale of NPS in head shops implied them as safe to consume, the PSA-initiated closure of these establishments consequently drove NPS sales to the streets and in turn made them more accessible to the most vulnerable (Haden et al., 2017).

### Limitations

As detection methods for NPS have advanced, and requests for NPS toxicology tests to be performed have become more frequent, part of the increase in NPSAD reporting over time is potentially an artefact of concomitant improvements in NPS detection methods (Ford and Berg, 2018; May et al., 2019; Mollerup et al., 2017; Segawa et al., 2019; Wagmann and Maurer, 2018). However, as standard toxicology screens do not include NPS, and even when requested different toxicology laboratories test against their own bespoke libraries within which there are detection limitations, the occurrence of deaths with NPS detected is likely under-reported (Wagmann and Maurer, 2018). Furthermore, as NPSAD is reported to voluntarily and coronial investigations are not carried out for all deaths, the figures presented here likely under-represent the true number of deaths where NPS had been consumed prior to death occurring in England, Wales and Northern Ireland.

Other UK drug policy changes during the post-PSA period may also have influenced drug use behaviours. For example, some of the substances classed as NPS in this study were controlled under the MDA in the post-PSA period. However, introduction of these subsequent MDA controls did not alter trends in the reporting of deaths where NPS were detected. Indeed, introduction of the PSA itself does not appear to have impacted upon NPS health risk awareness, or NPS drug demand (Deligianni et al., 2020). Increasingly risky drug-taking behaviours (UN, 2019) and societal changes may also have influenced patterns in NPS use. However, deprivation scores of neighbourhoods remained largely unchanged over the course of the study period (Ministry of Housing, Communities & Local Government, 2019), nor were there significant changes in the homeless or prison populations (Ministry of Housing, Communities & Local Government, 2021, Sturge, 2020). It is clearly evident however that the proportion of individuals in these subgroups who use NPS has increased over the duration of the study (Blackman and Bradley, 2017; Ford and Berg, 2018; Norman et al., 2020; Peacock et al., 2019; Scourfield et al., 2019; Yoganathan et al., 2021).

## Conclusions

Deaths with NPS detected continue to rise despite introduction of the PSA, and in many cases after their specific control under the MDA, further supporting evidence that current UK drug legislation approaches are not driving changes in NPS use behaviours (Deligianni et al., 2020). The relationship between the PSA and the displacement or replacement of NPS by established drugs of abuse needs further research. Whilst legality may not necessarily be a factor informing drug using behaviours, the PSA’s impact on price, and availability of NPS warrant further research into the relationship between MDA-controlled and PSA-controlled drug use. Notwithstanding, the PSA and MDA have worked together to reduce deaths amongst younger individuals living in more affluent areas, however it is clear that additional measures to prohibition are needed to curb their persistence in deprived demographics. Efforts to understand drug use as a disease rather than a crime to develop prevention, treatment and reintegration programmes to achieve drug-related harm reduction, as seen in Portugal, should be considered by UK policy makers (Cowan, 1986; Félix and Portugal, 2017).
